# Biodiversity of Endophytic Microbes in Diverse Tea Chrysanthemum Cultivars and Their Potential Promoting Effects on Plant Growth and Quality

**DOI:** 10.3390/biology12070986

**Published:** 2023-07-11

**Authors:** Tong Sun, Yanrong Yang, Kuolin Duan, Yuan Liao, Zhi Zhang, Zhiyong Guan, Sumei Chen, Weimin Fang, Fadi Chen, Shuang Zhao

**Affiliations:** 1 College of Horticulture, Nanjing Agricultural University, Nanjing 210095, China; 2020804251@stu.njau.edu.cn (T.S.); 2022104124@stu.njau.edu.cn (Y.Y.); 2020804250@stu.njau.edu.cn (K.D.); liaoyuan@njau.edu.cn (Y.L.); guanzhy@njau.edu.cn (Z.G.); chensm@njau.edu.cn (S.C.); fangwm@njau.edu.cn (W.F.);; 2 Key Laboratory of Landscaping, Ministry of Agriculture and Rural Affairs, Nanjing 210095, China; 3 Key Laboratory of Biology of Ornamental Plants in East China, National Forestry and Grassland Administration, Nanjing 210095, China; 4 Zhongshan Biological Breeding Laboratory, No.50 Zhongling Street, Nanjing 210014, China

**Keywords:** tea chrysanthemum, endophytes, microbial community composition, plant growth-promoting rhizobacteria

## Abstract

**Simple Summary:**

‘Chujv’, ‘Fubai jv’, ‘Hangbai jv’, ‘Jinsi huangjv’, and ‘Nannong jinjv’ are the five most popular tea chrysanthemum cultivars in China. Sustained monoculture often leads to plant growth inhibition and quality deterioration, causing significant economic losses to farmers. Endophytic microbes play pivotal roles in plant growth, development, and diversification. However, the effects of endophytes in various tea chrysanthemum cultivars under field conditions remain unknown. In the present study, the community composition and diversity of endophytic bacteria and fungi in five commercially popular tea chrysanthemums were characterized using high-throughput sequencing and culture-dependent methods. The endophytic microbiomes significantly differed across tea chrysanthemum cultivars and organs (stems and leaves). Importantly, it was indicated by the field study that 4 of 42 isolated endophytes could significantly promote tea chrysanthemum yield. These findings offer novel insights into the endophytic microbiomes of tea chrysanthemums and their potential applications in sustainable cultivation.

**Abstract:**

The endophytic microbiomes significantly differed across tea chrysanthemum cultivars and organs (stems and leaves). The most abundant endophytic bacterial genera were *Pseudomonas*, *Masillia*, and *Enterobacter* in the leaves and *Sphingomonas* and *Curtobacterium* in the stems of the five cultivars. Meanwhile, the most abundant endophytic fungal genera in the leaves and stems of the five tea chrysanthemums were *Alternaria*, *Cladosporium*, and *Sporobolomyces*. Specifically, *Rhodotorula* was dominant in the leaves of ‘Jinsi huangjv’ and *Paraphoma* was dominant in the stems of ‘Jinsi huangjv’. In all cultivars, the diversity and richness of endophytic bacteria were higher in leaves than in stems (*p* < 0.05). The highest diversity and richness of endophytic bacteria were recorded in ‘Chujv’, followed by ‘Jinsi huangjv’, ‘Fubai jv’, ‘Nannong jinjv’, and ‘Hangbai jv’. Meanwhile, endophytic fungi were less pronounced. Twenty-seven and 15 cultivable endophytic bacteria and fungi were isolated, four isolated endophytic bacteria, namely, CJY1 (*Bacillus oryzaecorticis*), CY2 (*Pseudomonas psychrotolerans*), JSJ7, and JSJ17 (*Enterobacter cloacae*) showed higher indole acetic acid production ability. Further field studies indicated that inoculation of these four endophytic bacteria not only promoted plant growth and yield but also increased total flavonoids, chlorogenic acid, luteolin, and 3,5-dicoffeylquinic acid levels in the dry flowers of tea chrysanthemums.

## 1. Introduction

Chrysanthemum (*Chrysanthemum morifolium* Ramat.) is a dicotyledonous genus in the family Asteraceae, which is well-known for its high ornamental and tea value [1]. The chrysanthemum originated in China. Over 20 cultivars of tea chrysanthemum are grown in different geographical regions of China, and the most popular cultivars include ‘Chujv’ (Chuzhou, Anhui), ‘Fubaijv’ (Machen, Hubei), ‘Hangbaijv’ (Tongxiang, Zhejiang), and ‘Jinsi huangjv’ (Xiushui, Jiangxi). With comprehensive research, the chemical components and pharmacological effects of various tea chrysanthemum cultivars have been revealed. Modern medicine studies have shown that chrysanthemum flowers are rich in bioactive constituents with numerous health benefits, such as protecting the liver, being anti-tumor, being anti-inflammatory, and producing anti-bacterial effects [2]. Thus, demand from consumers for tea chrysanthemums is expanding rapidly from year to year. However, with the increasing number of continuous cropping years, tea chrysanthemums often suffer from soil-borne diseases [3], resulting in a decline in soil fertility and an imbalance of soil microbial communities, which greatly restricts the yield and quality of tea chrysanthemums [4,5]. Chemical control is a commonly used strategy for disease control. However, it is not environmentally friendly and produces negative effects on beneficial microbes [6,7]. Therefore, the importance of biological control has increased over the years, and biocontrol has become a promising way to control diseases and increase plant yield and quality [8].

Endophytes are non-pathogenic micro-organisms that are widespread among plants and colonize the inter- and intracellular spaces of nearly 300,000 plant compartments [9]. The endophytic microbiome can be transmitted vertically from the seeds of the mother generation [10] or horizontally from the rhizosphere soil to the interior of the roots (endosphere) [11]. Horizontally transmitted endophytes enter the roots via adhesion to root cells using flagella, pili, exopolysaccharides, and hyphae [12]. The interactions between endophytic microbes and plants range from mutualism to latent pathogenicity, in which the plant provides a protective niche and endophytic microbes produce various useful metabolites that enhance nutrient uptake [13], further affecting plant growth and development [14,15].

The promotion of plant growth by plant growth-promoting rhizobacteria may be realized through the synthesis of plant hormones, such as indole-3-acetic acid (IAA), secretion of siderophores for iron acquisition, phosphate solubilization, nitrogen fixation, or disease suppression [13,16,17]. To date, diverse endophytes, including *Bacillus*, *Pseudomonas*, *Trichoderma*, *Pantoea*, and *Enterobacter* species, have been isolated and identified from various plants [18], and their pivotal roles in plant growth and stress response have been proven [19]. In particular, some endophytic *Bacillus* and *Pseudomonas* species have been shown to improve pathogen resistance of rice by stimulating induced systemic resistance.

As a new microbial resource, endophytes have received substantial attention in recent years, and their diversity and composition have become vital factors affecting plant productivity and health. Moreover, recent studies have provided novel insights into the diversity of endophytic microbial communities related to plant genotypes, plant compartments, host biogeography, plant growth stages, and seasons [20,21]. Although the community composition and diversity of microbiomes in the rhizosphere soil of pathogen-infected and healthy chrysanthemums and the effects of chrysanthemum cultivation on the soil microbiome have been explored [2,22], the endophytic microbial communities associated with tea chrysanthemums under different field locations, in various cultivars, and in diverse organs at the flowering stage remain to be extensively characterized.

Due to the unknown growth requirements of many microbes and the presence of cells that are in a viable but non-cultivable state, the portion of microbial diversity obtained using conventional cultivation techniques is less than 1% of the bacterial species present, and most information on the community and diversity of endophytic microbes has been obtained using culture-dependent approaches in recent years.

In the present study, cultural-dependent and amplicon sequencing-based methods were combined to characterize the endophytic microbial communities and diversity in five tea chrysanthemum cultivars and to isolate plant growth-promoting endophytes. The specific aim of the present study was to obtain comprehensive and accurate information on the diversity and community composition of endophytes associated with tea chrysanthemums and lay the practical foundation for green and efficient tea chrysanthemum cultivation using biocontrol resources.

## 2. Materials and Methods

### 2.1. Plant Material

Five tea chrysanthemum cultivars, namely, ‘Chujv’ (Chuzhou, Anhui), ‘Fubaijv’ (Machen, Hubei), ‘Hangbai jv’ (Jiaxing, Zhejiang), ‘Jinsi huangjv’ (Xiushui, Jaingxi), and ‘Nannong jinjv’ (Nanjing, Jiangsu), were collected from the major production district. Six healthy plants of each tea chrysanthemum cultivar were randomly sampled; stems (S) and six pieces of leaves (L) from each cultivar were collected, placed in different plastic bags, and transported to the laboratory at 4 °C.

### 2.2. DNA Extraction and PCR Amplification

Microbial genomic DNA was extracted using the HiPure Stool DNA Kit (Magen, Guangzhou, China), according to the manufacturer’s protocol. The concentration and integrity of the resulting DNA were determined using Aligo 2100. The V5–V7 region of the bacterial 16S rRNA was amplified using the gene-specific primers 799F and 1193R [23], and the ITS1 region of fungi was targeted using the primer sets KYO2F and ITS86R [24]; each sample was amplified with six replicates. All reactions were performed in a total volume of 50 μL, containing 10 μL of 5× Q5@ reaction buffer, 10 μL of 5× Q5@ High GC enhancer, 1.5 μL of 2.5 mM dNTPs, 1.5 μL of each primer (10 μM), 0.2 μL of Q5@ High-Fidelity DNA Polymerase, and 50 ng of template DNA, raised to 50 μL. Thermal cycling conditions were as follows: 95 °C for 5 min, followed by 30 cycles at 95 °C for 1 min, 60 °C for 1 min, and 72 °C for 1 min, and final extension at 72 °C for 7 min.

### 2.3. Illumina Sequencing

Amplicons were assessed in a 2% agarose gel and purified using the AxyPrep DNA Gel Extraction Kit (Axygen Biosciences, Union City, CA, USA), according to the manufacturer’s instructions. The purified amplicons were pooled at equimolar amounts and paired-end sequenced on an Illumina platform, following the standard protocols. All library preparations were performed on the Illumina Novaseq 6000 sequencing platform at Genedenovo Biotechnology Co., Ltd. (Guangzhou, China).

### 2.4. Data Analysis

Low-quality reads were filtered using FASTP (version 0.18.0) and assembled. Double-ended reads were spliced into tags using FLASH (version 1.2.11), and the tags were filtered using the UPARSE algorithm of USEARCH to obtain clean tags. Chimeric tags were removed using the UCHIME algorithm, and the tags were clustered into operational taxonomic units (OTUs) at ≥97% similarity using the UPARSE (version 9.2.64) pipeline. Based on OTU abundance information and species annotation information, the representative sequences were selected and further annotated with the database using the Naïve Bayesian Assignment algorithm of RDP Classifier (confidence threshold = 0.8–1.0).

Alpha-diversity analyses, including community richness (Chao and Ace indices) and community diversity (Shannon and Simpson indices) analyses, were performed using QIIME (version 1.9.1). Principal co-ordinate analysis (PCoA) was performed. Venn analysis was performed using the Venn diagram package of R (version 1.6.16) and the UpSetR package of R (version 1.3.3). Kyoto Encyclopedia of Genes and Genomes (KEGG) metabolic pathway analysis of bacteria was performed using PICRUSt (version 2.1.4), and functional groups of fungi were inferred using FUN Guild (version 1.0) [25].

### 2.5. Isolation and Identification of Endophytic Bacteria and Fungi

The stems and leaves of tea chrysanthemums were washed with tap and deionized water multiple times. Then, 5 cm stem segments and leaves from each plant were removed and immersed in 70% alcohol for 30 s, followed by transfer to 2% NaClO_3_ for 2 min. After disinfection, the surfaces were washed with sterile water for 2 min. The last rinse water (200 μL) was applied on the LB or PDA medium as control, which yielded no bacterial and fungal colonies when incubated at 28 °C for 3 days. The sterilized stems were cut into 0.5 cm segments and the leaves were cut into 0.5 cm squares (with wounds all around). The sampled stem phloem and leaves were inoculated onto separate media (PDA for endophytic fungi and LB for endophytic bacteria) and incubated at 28 °C for 3–6 days. Genomic DNA of endophytic microbes was extracted following the standard procedure [24]. ITS1F/ITS4R and 357F/518R were used to amplify the ITS region of fungi and the 16S rRNA genes of bacteria. The obtained 16S rRNA and ITS sequences of the endophytic bacterial and fungal strains were compared using the NCBI GenBank (http://www.ncbi.nlm.nih.gov/), accessed on 6 July 2021 and EzTaxon (https://www.ezbiocloud.net/) databases, accessed on 7 July 2021. A phylogenetic tree was constructed by the neighbor-joining method in Mega7.

### 2.6. Determination of IAA Content in the Fermentation Broth of Endophytic Bacteria

The strains were inoculated in Luria–Bertani (LB) medium with three replicates. The stains were incubated for 2 days at 37 °C while shaking at 200 rpm; a mixture of the same volume of uninoculated medium and colorimetric solution was used at the control. The fermentation broth of endophytic microbes was centrifuged for 10 min at 10,000 rpm; then, 200 μL of Salkowski display agent was added to 100 μL of the supernatant. IAA content of the supernatant was quantified based on pink color development after 30 min of incubation in the dark at 28–30 °C. A standard curve for IAA was constructed, and the IAA content for each strain was calculated [26].

### 2.7. Field Experiment Design and Inoculation of IAA-Producing Endophytic Stains

Experiments were conducted at the Chrysanthemum Germplasm Resource Preserving Center (Nanjing, China). Soil pH was 6.25; specific conductance was 391.27 μs·cm^−1^; organic matter content was 10.98 g·kg^−1^; and available nitrogen, phosphorus, and potassium contents were 98, 37, and 181 mg·kg^−1^, respectively. Before being transplanted to the field, cuttings of the tea chrysanthemum cultivar ‘Hongxin jv’ provided by Hexiang Chrysanthemum Modern Agricultural Industrial Park Co., Ltd. (Nanjing, China) were first established through culturing in perlite for 20 days in a greenhouse under a 16 h photoperiod and 70% relative humidity. The day and night temperatures were maintained at 28 °C and 22 °C, respectively. A 15-plot experiment was set following a randomized complete block design with three replicates and five treatments. Each plot measured 1.6 m × 0.4 m and was planted with 100 rooted cuttings. The inoculum concentration of the IAA-producing endophytic stains was 1.0 × 10^8^ per gram of soil.

### 2.8. Measurement of Plant Growth and Quality

Shoot height and diameter, shoot dry weight, leaf width and length, fresh and dry root weight, flower diameter, ray floret number, and yield were recorded; 12 plants were randomly sampled from each replicate during the seedling, budding, and flowering stages. Total flavonoids, chlorogenic acid, luteolin, and 3, 5-dicoffeylquinic acid levels in the flowers of tea chrysanthemums were measured [2]. Total flavonoids were extracted by 30 min by constant temperature ultrasound in a water bath of 70% ethyl alcohol at 60 °C. AL(NO_3_)3-NaNO_2_ spectrophotometric colorimetry was used. The content was measured with an enzyme-labeled assay and repeated three times for each sample. Chlorogenic acid, luteolin, and 3, 5-dicaffeoyl quinic acid were extracted by 70% HPLC-grade methyl alcohol and 30 min by constant temperature ultrasonic in a water bath at 60 °C. The chromatography was performed on Rapid Resolution (4.6 × 100 mm, 3.5 μm) column with acetonitrile as mobile phase A and 0.1% phosphoric acid solution as mobile phase B. The column temperature was 30 °C, the flow rate was l.0 mL/min, and the detection wavelength was 348 mn. The peak area was determined by HPLC, and the standard samples of chlorogenic acid, luteolin, and 3, 5-dicaffeoyl quinic acid were used for quantitation. Each sample was repeated three times.

## 3. Results

### 3.1. Composition of Endophytic Microbiomes

**Figure 1:**
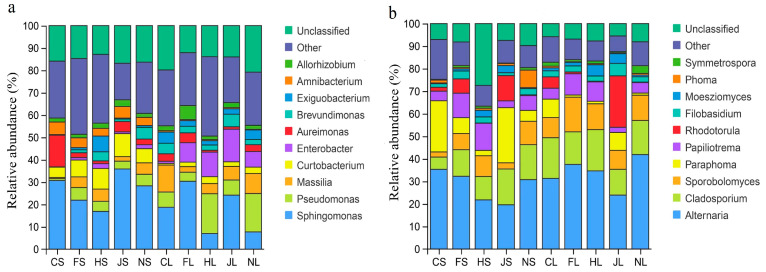
Composition and relative abundance of endophytic bacteria (**a**) and fungi (**b**) in different samples at the genus level. The color of the column represents different genera, and the length of the column represents the proportion of the genus. Sequences that could not be classified into any known group were considered unclassified. Genera that comprised <1% of the total composition of each sample were classified as other genera. Note: CS, FS, HS, JS, NS, CL, FL, HL, JL, and NL were the abbreviations of ‘Chujv’ stem, ‘Fubai jv’ stem, ‘Hangbai jv’ stem, ‘Jinsi huangjv’ stem, ‘Nannong jinjv’ stem, ‘Chujv’ leave, ‘Fubai jv’ leave, ‘Hangbai jv’ leave, ‘Jinsi huangjv’ leave, and ‘Nannong jinjv’ leave, respectively.

The major endophytic bacteria and fungi detected in the different organs of the five tea chrysanthemum cultivars were analyzed at the genus level (Figure 1). The relative abundance of the same genera varied among the different tea chrysanthemum samples. Among the endophytic bacteria, the top 12 genera with >1% abundance were *Allorhizobium*, *Amnibacterium*, *Exiguobacterium*, *Brevundimons*, *Aureimonas*, *Enterobacter*, *Masillia*, *Pseudomonas*, and *Sphingomonas*. *Sphingomonas* (5.69~35.82%) and *Curtobacterium* (4.15–10.08%) were the two most abundant endophytic bacterial genera in stem samples (Figure 1a). *Pseudomonas*, *Masillia*, *Enterobacter*, and *Exiguobacterium* were detected more frequently in leaves, while *Aureimonas* (1.98–16.15%) and *Amnibacterium* (3.62–6.89%) were found mostly in stems. Among endophytic fungi, the top 12 genera with >1% abundance were *Alternaria*, *Cladosporium*, *Sporobolomyces*, *Paraphoma*, *Papilotrema*, *Rhodotorula*, *Falobasidium*, *Moesziomyces*, *Phoma*, and *Symmetrospora* (Figure 1b). *Alternaria* (19.16–42.17%), *Paraphoma* (1.28–24.25%), and *Sporobolomyces* (3.26–15.24%) were found in most tested samples. The highest abundance of *Alternaria* (42.23%) was recorded in NL, followed by FL and CS. The highest abundance of *Rhodotorula* (23.51%) was recorded in JL and that of *Papilotrema* (12.39%) was recorded in HS.

### 3.2. Diversity of Endophytic Microbiome

**Figure 2:**
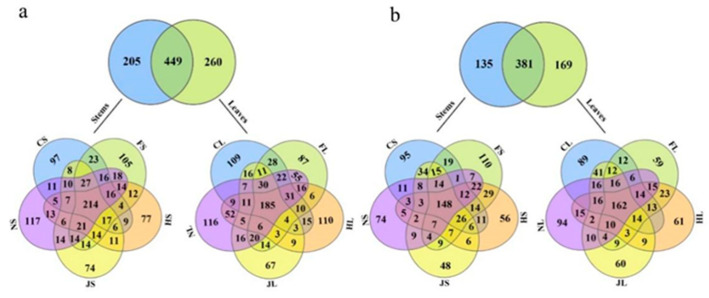
Venn diagrams showing shared unique OTUs of endophytic bacteria (**a**,**b**) fungi in the stem and leaf samples of five tea chrysanthemum cultivars. Note: The shown numbers in the figures are unique OTUs against total number of OTUs found through all samples.

The alpha diversity analyses of endophytic bacteria and fungi were performed using OTUs versus sequences obtained from each tea chrysanthemum cultivar and organ. Venn diagrams showed that the stems and leaves of the five tea chrysanthemum cultivars harbored, respectively, 205 and 260 unique endophytic bacterial OTUs and shared 449 OTUs (Figure 2a). There were 97, 105, 77, 74, and 117 unique OTUs and 214 shared OTUs in the stem samples of CS, FS, HS, JS, and NS, respectively, and 109, 87, 110, 67, and 116 unique OTUs and 185 shared OTUs in the leaf samples of CL, FL, HL, JL, and NL, respectively. Moreover, there were 135 and 169 unique and 381 shared endophytic fungal OTUs in the stem and leaf samples (Figure 2b). The stem samples of CS, FS, HS, JS, and NS harbored, respectively, 95, 110, 56, 48, and 74 unique OTUs and 148 shared OTUs, whereas the leaf samples of CL, FL, HL, JL, and NL harbored, respectively, 89, 59, 61, 60, and 94 unique OTUs and 162 shared OTUs. The number of endophytic bacteria and fungi in the leaves was higher than that in the stems and the number of unique OTUs of endophytic bacteria and fungi was the highest in NS and FS, respectively.

The highest richness (Chao1 and Ace indices) of endophytic bacteria in stems was recorded in CS, whereas the lowest richness was recorded in HS. The highest richness of endophytic bacteria in leaves was recorded in CL, followed by JL and FS. The diversity (Shannon and Simpson indices) of endophytic bacteria varied among different cultivars and organs. The highest Shannon index was recorded in CL (Table 1), indicating that leaves harbored the highest endophytic bacterial community diversity. For endophytic fungi, the highest richness in stem samples was recorded in HS, followed by JS and CS. The highest richness of endophytic fungi in leaves was recorded in JL, although there were no significant differences among the other groups.

**Figure 3:**
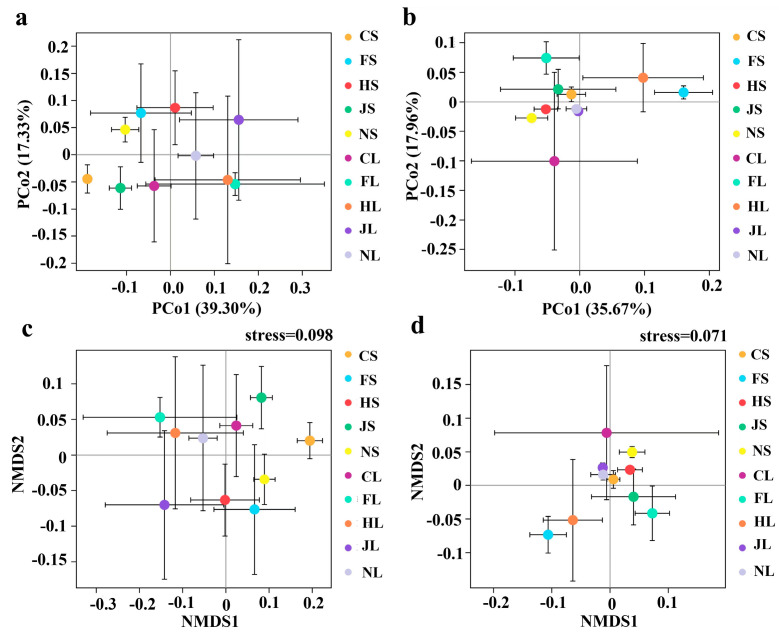
PCoA diagram and NMDS of endophytic bacterial (**a**,**c**) and fungal (**b**,**d**) communities and in the stems and leaves of tea chrysanthemum cultivars.

PCoA graphically revealed significant differences in endophytic bacterial and fungal communities across different tea chrysanthemum cultivars and organs. For endophytic bacteria, the weighted UniFrac distances showed that all samples were clearly separated from one another along the first co-ordinate axis (PCA1), except for HL and FL (ANOSIM, CL vs. NL vs. JL vs. HS vs. FS vs. CS vs. NS, *p* < 0.001), whereas HL and FL were tightly grouped together based on the weighted UniFrac distances and non-metric multi-dimensional scaling analysis (NMDS) (Figure 3a,c). For endophytic fungi, HS, JL, JS, and CS showed significant differences based on weighted UniFrac distances (ANOSIM, HS vs. JL vs. JS vs. CS, *p* < 0.001) along PCA1 and PCA2, whereas NL and NS were grouped closely together based on weighted UniFrac distances and NMDS (ANOSIM, HS vs. JL vs. JS vs. CS, *p* < 0.001) (Figure 3b,d).

### 3.3. Gene-Oredicted Functional Profiles of Endophytic Bacterial and Fungi

**Figure 4:**
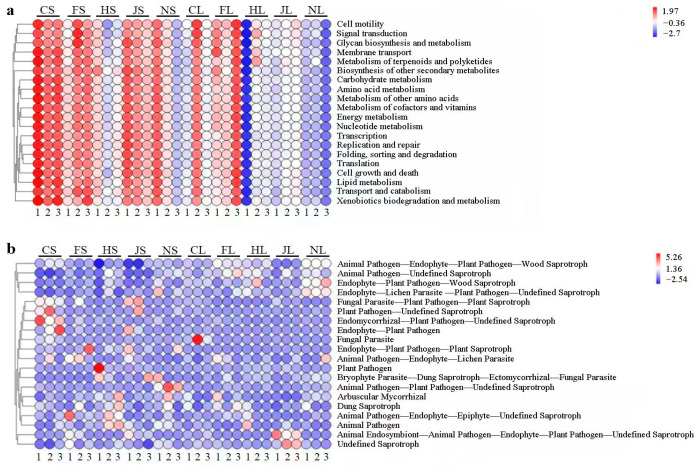
Gene-predicted functional profiles of endophytic bacteria (**a**) and fungi (**b**) obtained using PICRUSt2 and FUN Guild. Red and blue represent high and poor enrichment in functional abundance, respectively.

Twenty level 2 KEGG Orthology (KO) groups were detected. Functions related to cell motility, signal transduction, glycan biosynthesis and metabolism, membrane transport, biosynthesis of other secondary metabolites, metabolism of terpenoids and polyketides, carbohydrates, ammonia acids, cofactors and vitamins, energy, nucleotides, transcription, replication and repair, lipids, folding, sorting and degradation, cell growth and death, transport and catabolism, xenobiotic biodegradation, and metabolism of soil endophytic bacteria were significantly enriched in CL. Meanwhile, ITS gene-predicted functional profiles based on PICRUSt2 inference revealed that the functions of soil endophytic fungi associated with animal pathogen–endophytes–plant pathogen–wood saprotroph, animal pathogen–undefined saprotroph, endophyte–plant pathogen–wood saprotroph, and endophyte–lichen parasite–plant pathogen–undefined saprotroph were significantly suppressed in CS. Moreover, the functions of soil endophytic fungi associated with parasite–plant pathogen–plant saprotroph, plant pathogen–undefined saprotroph, endomycorrhizal–plant pathogen–undefined saprotroph were obviously enriched in CL (Figure 4b). Overall, the number of fungal pathways was significantly lower than that of bacterial pathways in all samples. Nutrient types in soil fungal communities were primarily pathologically parasitic and saprophytic.

### 3.4. Composition of Endophytic Bacterial and Fungal Communities Based on the Culture-Dependent Method

**Figure 5:**
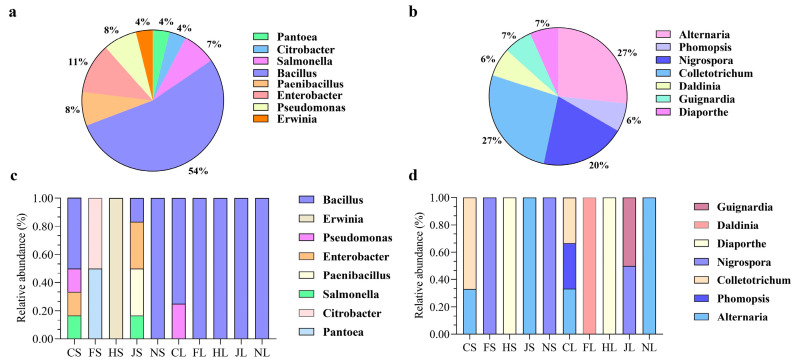
Distribution and relative abundance at the genus level of endophytic bacteria (**a**,**c**) and fungi (**b**,**d**) in the stems and leaves of five tea chrysanthemum cultivars.

A total of 27 cultivable endophytic bacteria and 15 cultivable endophytic fungi were isolated from the sterilized stems and leaves of the five tea chrysanthemum cultivars. Endophytic bacteria were classified into two different phyla (*Proteobacteria*, 38%, and *Firmicutes*, 62%), eight genera, and 27 species, and endophytic fungi were classified into two phyla (*Deuteromycotina*, 53%, and *Ascomycota*, 47%), seven genera, and 15 species. The distribution of endophytic bacterial genera in all collected samples was 54% *Bacillus*, 8% *Paenibacillus*, 11% *Enterobacter*, 8% *Pseudomonas*, 7% *Salmonella*, and 4% each *Pantoea*, *Erwinia*, and *Citrobacter* (Figure 5a). Meanwhile, the distribution of endophytic fungal genera was 27% each *Colletotrichum* and *Alternaria*, 20% *Nigrospora*, 7% each *Diaporthe*, 7% *Daldinia*, and *Guignardia*, and 6% *Phomopsis* (Figure 5b). The relative abundances of *Bacillus*, *Enterobacter*, and *Salmonella* were, respectively, 52%, 17%, and 18.5% in CS and, respectively, 19%, 34%, and 17% in JS. Moreover, the abundance of *Pseudomonas* was 12.5% in CS, and that of *Paenibacillus* was 29% in JS (Figure 5c). The relative abundances of the isolated endophytic fungal genera *Alternaria*, *Phomopsis*, and *Colletotrichum* were, respectively, 31.50%, 41.60%, and 26.90% in CS (Figure 5d). The abundance of *Bacillus* was 80% and that of *Pseudomonas* was 20% in CL. The distribution of cultivable endophytic bacterial and fungal communities varied across cultivars and organs. The diversity of endophytic bacteria in stems was greater than that of leaves.

### 3.5. Selection and Identification of IAA-Producing Endophytic Strains

**Figure 6:**
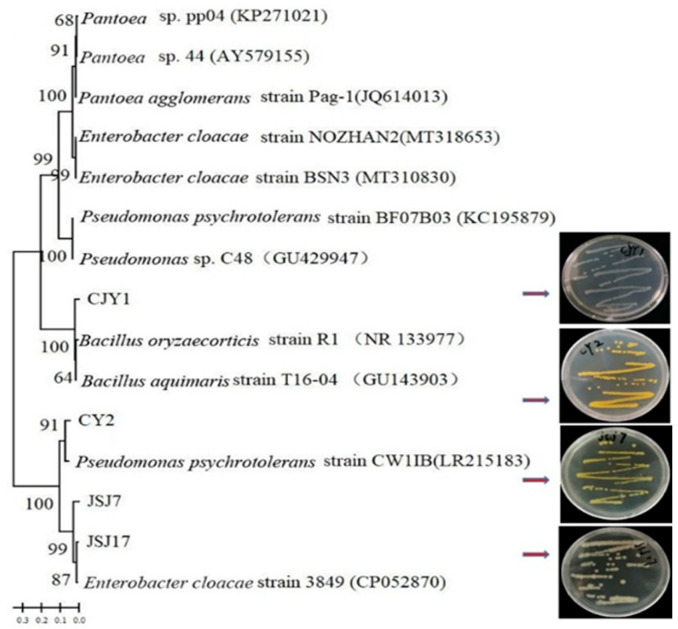
Neighbor-joining phylogenetic tree based on 16s rDNA genes sequence of CJY1, CY2, JSJ7, and JSJ17.Note: At the nodes, bootstrap values greater than 60 percent (reported as percentages of 1000 replications) are provided.

The IAA-producing abilities of 42 species were tested using the Salkowski method. Thirteen endophytic bacteria showed IAA-producing abilities, and their IAA contents ranged from 3.5 to 118.59 μg·mL^−1^. Among these, the IAA contents of JSJ7 (118.59 μg·mL^−1^), JSJ17 (72.44 μg·mL^−1^), CJY (82.83 μg·mL^−1^), and CY2 (54.08 μg·mL^−1^) were higher than those of the other isolates (data not shown). Moreover, these four IAA-producing endophytes grew well on LB solid medium. CJY1 colonies were white, transparent, and round, with bulging surfaces and smooth edges. CY2 colonies were yellow and opaque, with rough and ruffled surfaces, and unsmooth edges. JSJ7 colonies were yellow, opaque, and round, with smooth surfaces and regular edges. JSJ17 colony was opacified and opaque, with a protuberant surface and irregular edges (Figure 6). These four strains were identified as *Bacillus oryzaecorticis* (CJY1), *Pseudomonas psychrotolerans* (CY2), and *Enterobacter cloacae* (JSJ7and JSJ17) (Figure 6).

### 3.6. Effect of IAA-Producing Endophytic Bacterial Inoculation on Plant Growth and Quality

Inoculation of the IAA-producing endophytic strains CJY1, CY2, JSJ7, and JSJ17 significantly affected the growth of ‘Hongxin jv’. Specifically, CJY1, CY2, JSJ7, and JSJ17 inoculation significantly increased shoot height as well as leaf, root, and flower indices. At the seedling stage, the highest indices of shoot height, crown diameter, leaf fresh weight, and leaf dry weight were recorded in the JSJ7 treatment, with increases of, respectively, 36.19%, 34.82%, 78.96%, and 84.14%, compared with values in the CK treatment. The highest stem diameter was recorded in the CJY1 treatment, with an increase of 97.08% compared with value in the CK treatment (Table 2). At the budding stage, stem diameter increased by, respectively, 12.16%, 11.99%, 15.67%, and 14.48% and the crown diameter increased by, respectively, 10.67%, 24.82%, 42.24%, and 35.20% in the CJY1, CY2, JSJ7, and JSJ17 treatments compared with values in the CK treatment. Higher fresh and dry weights of stems and leaves were recorded in the JSJ7 treatment, and both JSJ7 and CY2 treatments increased the dry weights of roots. At the flowering stage, there were no significant differences in stem diameter, root dry weight, stem fresh weight, and leaf dry weight among the treatments; however, shoot height, crown diameter, and root dry weight increased by, respectively, 43.77%, 34.00%, and 36.40% in the JSJ7 treatment (Table 2).

### 3.7. Effect of IAA-Producing Endophytic Bacterial Inoculation on Plant Yield and Quality

Inoculation of the four IAA-producing endophytic bacteria obviously improved the yield of ‘Hongxin jv’ (Table 3). The estimated yield of ‘Hongxin jv’ increased by, respectively, 145.51%, 120.21%, 149.49%, and 145.11%; flower diameter increased by, respectively, 10.99%, 12.40%, 12.31%, and 13.98%; and the number of florets increased by, respectively, 62.03%, 49.83%, 73.22%, and 49.14% in the CJY1, CY2, JSJ7, and JSJ17 treatments compared with values in the CK treatment. However, there were no significant differences in mean flower dry weight.

**Figure 7:**
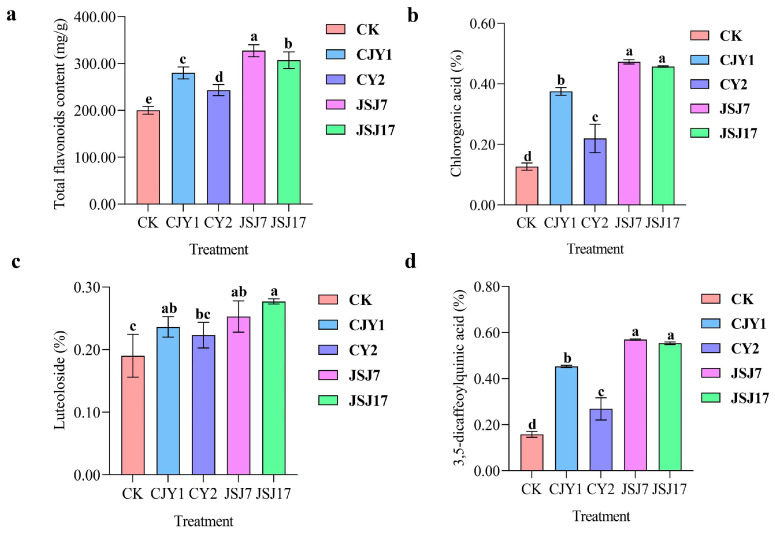
Effects of inoculation of the four IAA-producing endophytic bacteria on the secondary metabolites of tea chrysanthemum. Note: Data are presented as mean ± standard error; different lower-case letters in the same column indicate significant differences among the treatments (*p* < 0.05).

Compared with values in the CK treatment, the levels of total flavonoids (Figure 7a), chlorogenic acid (Figure 7b), luteoloside (Figure 7c), and 3, 5-dicoffeylquinic acid (Figure 7d) were significantly increased in the four IAA-producing endophytic bacterial inoculation treatments. The content of total flavonoids in the CJY1, CY2, JSJ7, and JSJ17 treatments was increased by, respectively, 39.87%, 21.51%, 63.51%, and 53.41%. The highest content of chlorogenic acid in the flowers was recorded in the JSJ7 treatment, which was significantly higher than that in the CJY1 and CY2 treatments. The content of 3, 5-dicafeylquinic acid was increased by, respectively, 21.05%, 31.58%, and 47.37% in the CJY1, JSJ7, and JSJ17 treatments compared to that in the CK treatment.

## 4. Discussion

### 4.1. Endophytic Community Composition and Diversity in Tea Chrysanthemum Cultivars

The present study demonstrated that the tea chrysanthemum microbiome differed across cultivars, consistent with previous reports in soybeans, ginseng (*Panax ginseng*), and Dendrobium [27,28,29]. Meanwhile, both bacterial and fungal community compositions shifted more across cultivars than across organs among the tested tea chrysanthemum samples, indicating that cultivar specificity is a strong selective force for microbial communities. These results are consistent with previous reports that the diversity of bacterial and fungal communities varies significantly across plant genotypes and that the plant compartments in a microhabitat facilitate the recruitment and establishment of different microbial communities in the endosphere [30]. Compared with stems, endophytic bacteria in leaves showed a higher diversity index, which may be because endophytic microbes generally originate from the rhizosphere or phyllosphere, enter the plant either through natural openings or wounds, and reach different plant tissues to establish themselves [31,32,33]. In addition, PCoA revealed that the composition of endophytic bacterial communities in the leaves and stems of the tested tea chrysanthemums were distinct from one another, while the components of endophytic fungal communities were grouped closely together, except in the stem of ‘Jinsi huangjv’, ‘Chujv’, and ‘Hangbai jv’, indicating that the diversity of bacteria was greater and more pronounced than that of fungi. This may be because the host genotype [34] and transgenic cultivars significantly affected the bacterial community composition in leaves but showed a weaker effect on communities in the roots. These differences in the extent of impact may be attributed to variations in the inoculation pools of microbes colonizing diverse habitats (i.e., leaves, stems, and pods have contact with air and rain, while roots have contact with soil), and the effects of tea chrysanthemum tissue niches on bacterial community composition indicate that microbial community differentiation is driven by biotic (plant selection) or abiotic (environment) factors.

Across the tea chrysanthemum organ compartments, we observed a significant contribution of the host genotype to the dominant genera of endophytic microbes. Among the endophytic bacteria, *Sphingomonas* and *Pseudomonas* were the two most frequently detected genera in the five tea chrysanthemum cultivars; *Pseudomonas* was more abundant in leaves, while *Curtobacterium* was more abundant in stems. *Aureimonas* was dominant in the stem of ‘Chujv’, while *Enterobacter* was dominant in the leaves of other cultivars. The host genotype explained more variation in the leaf bacterial community composition, whereas the genotype explained more variation in the leaf fungal community composition, followed by stems and roots. These results are consistent with previous reports [35,36,37,38].

By comparing the sequence data of the isolated endophytic bacteria, *Bacillus* was significantly enriched in stems among the five tea chrysanthemum cultivars, except in the stem of ‘Fubai jv’. However, *Sphingomonas* was highly abundant, as revealed by Illumina-based analysis. Previous studies have shown that *Bacillus* and *Sphingomonas* are common genera in plant tissues, with key roles in host metabolism and maintaining the stability of endophytic microflora [39,40]. Of note, *Sphingomonas* was predominant in the stems and leaves of the five tea chrysanthemum cultivars, whereas it could not be isolated with the culture-dependent method. Similarly, *Alternaria* was the most detected genus among the tested cultivars, which could not be isolated from the stem of ‘Fubai jv’, ‘Hangbai jv,’ and ‘Nannong Jingjv’ nor from the leaves of ‘Fubai jv’, ‘Hangbai jv’, and ‘Jinsi Huangjv’. Culture-dependent methods tend to underestimate the species and number of microbes present in plant tissues. Although Illumina-based analysis can easily detect more redundant genera, which is a more reliable method for studying plant endogenous microbial communities, the cultivable bacteria were probably the largest and most active bacteria in the tested tea chrysanthemums. In addition, the sequences of some clones showed low identity with the cultured bacterial or fungal genera but high identity with the uncultured bacteria and fungi, revealing the presence of some uncultured microbes in the tea chrysanthemum endophytic community.

### 4.2. Selection and Evaluation of Plant Growth-Promoting Endophytic Microbes

Research on the use of endophytes as bioinoculants as an alternative to conventional crop improvement in agriculture has yielded promising results [41]. The importance of microbes in plant growth promotion depends on their ability to fix nitrogen, synthesize indole-3-acetic acid, and produce siderophores, phytohormones, and antimicrobial compounds. In the present study, we isolated 42 endophytes from the stems and leaves of the five tea chrysanthemum cultivars tested. The majority of the 27 bacterial endophytes isolated belonged to the genera *Bacillus*, *Paenibacillus*, *Pseudomonas*, *Pantoea*, and *Enterobacter*. Importantly, many of the isolates were able to synthesize IAA; of these, four endophytic bacteria showed high IAA-producing ability, ranging from 54.08 to 118.59 μg·mL^−1^ (data not shown). Further field study demonstrated that *Bacillus oryzaecorticis* (CJY1), *Pseudomonas psychrotolerans* (CY2), and *Enterobacter cloacae* (JSJ7 and JSJ17) inoculation promoted tea chrysanthemum growth. These results corroborate previous findings that *Bacillus* and *Enterobacter* in soybean, *Pseudomonas* in maize, and some strains of *Pantoea* as endophytes in rice seeds could promote plant growth [42,43,44]. Additionally, some sequences showed high identity with *Citrobacter*, *Salmonella*, and *Erwinia*. To the best of our knowledge, these strains have not been previously observed as endophytic bacteria in chrysanthemum. According to the 2020 edition of the Pharmacopoeia of the People’s Republic of China, the chlorogenic acid and luteoloside contents in dried chrysanthemum should not be below 0.20% and 0.08%, respectively, and the content of 3, 5-dicaffeoylquinic acid content should not be below 0.70%. In the present study, the contents of chlorogenic acid and luteoloside in the dry flowers of tea chrysanthemum were <0.20% in the CK treatment. After inoculation of the four endophytic bacteria, the content of total flavonoid, chlorogenic acid, luteoloside, and 3, 5-dicaffeoylquinic acid in the dry flowers of tea chrysanthemum were significantly increased. These trends are consistent with the results of a previous study showing that diverse secondary metabolic products could be obtained from a wide range of genera, such as *Bacillus* and *Pseudomonas* [45], and that plant-associated microbes could influence important traits. For instance, these microbes produce a large amount of novel and bioactive secondary metabolites that are not only beneficial to the host plant but also economically important to humans for potential applications in pharmaceuticals and agriculture.

### 4.3. Interactions of Endophytic Microbes with Plant Cultivars and Their Promoting Effects on Plant Growth and Quality

**Figure 8:**
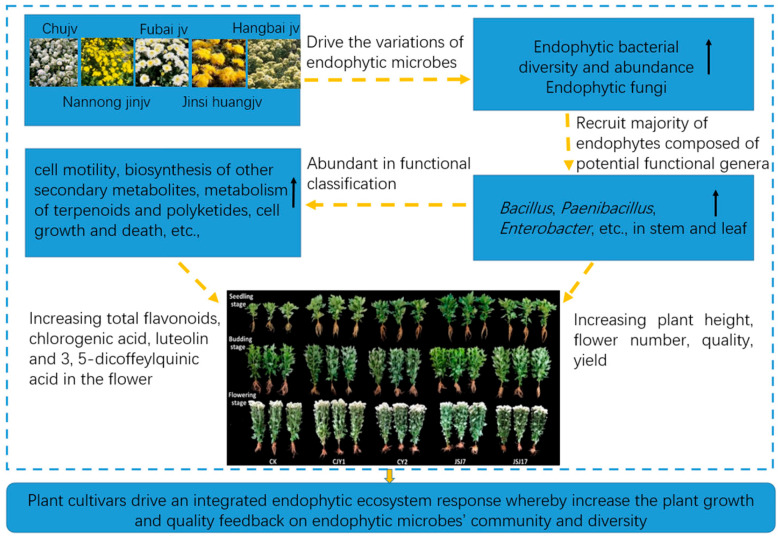
Conceptual diagram illustrating the mechanisms of tea chrysanthemum cultivars in shaping endophytic microbial community and PGPR in the growth and quality of the tea chrysanthemum.

Endophytes are important microbial resources that can maintain sustainable agriculture via numerous independent or linked mechanisms [46,47]. The root, stem, and leaf endospheric harbor diverse microbial communities that form close interactions with the host plant. Our results indicated that different tea chrysanthemum cultivars hosted highly diverse and complex endophytic microbial communities, and the diversity of the endophytic bacteria in the leaves was higher than that in the stems, implying that endophytic bacterial strains via active colonization mostly occupy the micro-niches within the tissues of tea chrysanthemums and possess ecological significance. Different tea chrysanthemum cultivars shelter various endophytes [48,49]. Our results showed that the microbial endophytic genera *Bacillus*, *Paenibacillus*, *Curtobacterium*, *Enterobacter*, *Pseudomonas*, *Erwinia*, *Citrobacter*, *Salmonella*, and *Pantoea* were recruited from different cultivars and organs, and some species showed plant growth-promoting abilities. To date, harnessing bio-incentives from microbial endophytes isolated from various plants and their application have contributed to crop yield. Therefore, we selected and identified four IAA-producing endophytic bacteria, namely, *Bacillus oryzaecorticis* (CJY1), *Pseudomonas psychrotolerans* (CY2), and *Enterobacter cloacae* (JSJ7 and JSJ17), which showed significant plant growth-promoting effects on tea chrysanthemums after inoculation. Endophytes in stems of ‘Chujv’, followed by the ‘Fubai jv’ and ‘Jinsi Huangjv’, were significantly enriched in functional classifications, such as “cell motility”, “signal transduction”, “glycan biosynthesis and metabolism”, “membrane transport”, “biosynthesis of other secondary metabolites”, “metabolism of terpenoids and polyketides”, “carbohydrates”, “ammonia acids”, “cell growth and death”, “transport and catabolism”, and “metabolism” (Figure 8).

These results are consistent with previous reports that endophytes present in medicinal or tea plants can affect plant growth directly via nitrogen fixation and growth-promoting stimulator production. IAA acts as an effector molecule in plant–microbe interactions. Our results also imply that endophyte infiltration is facilitated by exudate secretion containing signal molecules in exchange for nutrients in and out of the so-root environment [36,45,46]. The diverse endophytic micro-organisms occupying the external and intracellular compartments of plants play important roles in plant ecology and physiology, as diverse endophytic microbes inhabiting different plant organs may exhibit genetic relatedness. The exploration and application of endophytic micro-organisms in modern sustainable agriculture can guarantee maximum plant production and food safety. Moreover, research focusing on microbial bioinoculants as biofertilizers and biopesticides in sustainable agriculture will offer efficient and environmentally friendly solutions for green tea cultivation.

## 5. Conclusions

The present study investigated the endophytic bacterial and fungal communities in the stems and leaves of five popular tea chrysanthemum cultivars using a culture-dependent method and high-throughput sequencing. A rich diversity of endophytic bacteria was noted in the five tea chrysanthemum cultivars tested, and the distribution of these endophytic bacterial communities varied across organs and cultivars. Moreover, tea chrysanthemums could recruit a majority of the potentially functional endophytic genera, such as *Bacillus*, *Paenibacillus*, *Pseudomonas*, *Pantoea*, and *Enterobacter*, which have been shown to be candidates for biocontrol agents and growth inoculants, possibly benefitting tea chrysanthemums. Furthermore, our study clarified the factors affecting the community structure of endophytic bacteria and fungi in five tea chrysanthemum cultivars and laid the foundation for the use of endophytic microbes to promote the growth and improve the quality of tea chrysanthemums. In the future, the growth-promotion mechanism of the isolated endophytic bacteria in tea chrysanthemums warrants further research, and a culture-dependent method should be employed to screen endophytic bacteria and fungi with growth-promoting or biocontrol effects as an effective method for high yield and sustainable production of commercially important tea chrysanthemums.

## Figures and Tables

**Table 1:** Alpha diversity of endophytic bacteria and fungi in the stems and leaves of tea chrysanthemum cultivars.

Samples	Alpha Diversity Indices of Endophytic Bacteria				Alpha Diversity Indices of Endophytic Fungi			
	Chao 1	Ace	Simpson	Shannon	Chao 1	Ace	Shannon	Simpson
	619.86 ± 45.23 ^a^	620.85 ± 35.24 ^a^	5.51 ± 0.29 ^a^	0.94 ± 0.004 ^a^	426.26 ± 28.78 ^c^	416.89 ± 19.68 ^c^	3.34 ± 0.32 ^c^	0.86 ± 0.004 ^c^
FS	549.32 ± 25.12 ^c^	544.05 ± 27.12 ^c^	4.98 ± 0.15 ^c^	0.92 ± 0.007 ^c^	421.24 ± 32.56 ^c^	418.57 ± 21.58 ^c^	3.44 ± 0.25 ^c^	0.87 ± 0.004 ^c^
HS	496.90 ± 29.35 ^d^	498.47 ± 47.21 ^d^	4.24 ± 0.36 ^d^	0.90 ± 0.002 ^d^	457.10 ± 31.26 ^a^	443.66 ± 15.74 ^a^	4.86 ± 0.25 ^a^	0.80 ± 0.005 ^a^
JS	569.84 ± 15.28 ^b^	578.02 ± 38.12 ^b^	5.24 ± 0.35 ^b^	0.93 ± 0.005 ^b^	439.64 ± 19.56 ^b^	431.84 ± 15.2 ^b^	3.77 ± 0.18 ^b^	0.83 ± 0.003 ^b^
NS	502.56 ± 47.26 ^d^	510.24 ± 42.59 ^d^	4.51 ± 0.43 ^d^	0.90 ± 0.007 ^d^	424.14 ± 24.36 ^c^	420.84 ± 11.26 ^c^	3.39 ± 0.24 ^c^	0.86 ± 0.004 ^c^
CL	682.85 ± 51.23 ^a^	679.16 ± 58.42 ^a^	6.75 ± 0.31 ^a^	0.94 ± 0.006 ^a^	421.54 ± 17.56 ^b^	399.45 ± 34.57 ^b^	3.54 ± 0.17 ^b^	0.83 ± 0.003 ^b^
FL	516.82 ± 38.69 ^c^	514.21 ± 47.58 ^c^	5.29 ± 0.25 ^c^	0.91 ± 0.002 ^c^	418.42 ± 35.64 ^b^	415.83 ± 23.69 ^b^	3.49 ± 0.24 ^b^	0.84 ± 0.002 ^a^
HL	507.52 ± 34.89 ^c^	508.83 ± 41.29 ^c^	5.14 ± 0.27 ^c^	0.91 ± 0.005 ^c^	415.03 ± 23.56 ^b^	407.82 ± 31.56 ^b^	3.61 ± 0.29 ^b^	0.83 ± 0.003 ^b^
JL	575.55 ± 38.21 ^b^	545.07 ± 35.21 ^b^	5.98 ± 0.38 ^b^	0.93 ± 0.007 ^b^	453.50 ± 22.25 ^a^	449.65 ± 21.35 ^a^	3.93 ± 0.32 ^a^	0.80 ± 0.001 ^a^
NL	510.90 ± 59.32 ^c^	522.43 ± 54.67 ^c^	5.26 ± 0.47 ^c^	0.90 ± 0.007 ^a^	419.93 ± 21.25 ^b^	412.57 ± 20.45 ^b^	3.59 ± 0.33 ^b^	0.83 ± 0.001 ^b^

Note: Data are presented as mean ± standard error; different lower-case letters in the same column indicate significant differences among the treatments (*p* < 0.05).

**Table 2:** Effects of inoculation of IAA-producing endophytic bacteria on the growth of tea chrysanthemum.

		Shoot		Stem			Leave		Root
Growth Stages	Treatment	Height (cm)	Crown Diameter (cm)	Diameter (mm)	Fresh wt (g)	Dry wt (g)	Fresh wt (g)	Dry wt (g)	Dry wt (g)
Seedling stage (30 d)	CK	16.08 ± 1.08 ^c^	7.41 ± 13.4 ^b^	4.19 ± 0.35 ^b^	4.22 ± 1.09 ^c^	0.66 ± 0.11 ^c^	12.45 ± 2.92 ^c^	1.45 ± 0.28 ^c^	0.33 ± 0.63 ^b^
	CJY1	21.05 ± 1.12 ^ab^	9.57 ± 5.22 ^a^	5.05 ± 0.27 ^a^	8.04 ± 1.25 ^ab^	1.21 ± 0.18 ^ab^	21.08 ± 2.85 ^ab^	2.45 ± 0.32 ^ab^	0.38 ± 0.10 ^b^
	CY2	20.05 ± 1.10 ^b^	9.82 ± 8.52 ^a^	4.86 ± 0.48 ^a^	6.64 ± 0.95 ^b^	1.03 ± 0.15 ^b^	18.08 ± 1.47 ^b^	2.19 ± 0.23 ^b^	0.52 ± 0.12 ^a^
	JSJ7	21.90 ± 1.03 ^a^	9.99 ± 4.36 ^a^	4.96 ± 0.29 ^a^	8.03 ± 1.49 ^a^	1.34 ± 0.20 ^a^	22.28 ± 2.09 ^a^	2.67 ± 0.23 ^a^	0.58 ± 0.06 ^a^
	JSJ17	20.47 ± 0.84 ^b^	8.17 ± 8.61 ^b^	4.40 ± 0.30 ^b^	6.94 ± 1.06 ^b^	1.09 ± 0.13 ^b^	19.61 ± 1.68 ^ab^	2.36 ± 0.29 ^ab^	0.58 ± 0.08 ^a^
Budding stage (60 d)	CK	40.46 ± 1.93 ^b^	13.78 ± 29.7 ^c^	5.49 ± 0.78 ^b^	25.85 ± 5.54 ^c^	4.41 ± 0.68 ^c^	53.52 ± 5.54 ^d^	6.38 ± 0.60 ^c^	1.02 ± 0.19 ^b^
	CJY1	45.38 ± 1.47 ^a^	15.25 ± 17.3 ^bc^	6.42 ± 0.71 ^a^	35.24 ± 3.93 ^b^	6.60 ± 0.82 ^b^	69.20 ± 0.19 ^c^	8.63 ± 0.68 ^b^	1.01 ± 0.95 ^b^
	CY2	45.31 ± 1.81 ^a^	17.20 ± 5.87 ^ab^	6.77 ± 0.32 ^a^	37.26 ± 2.83 ^ab^	7.55 ± 0.68 ^ab^	73.85 ± 3.61 ^bc^	9.84 ± 0.76 ^ab^	1.61 ± 0.20 ^a^
	JSJ7	46.80 ± 2.20 ^a^	19.60 ± 0.18 ^a^	6.89 ± 0.16 ^a^	43.80 ± 5.58 ^a^	8.19 ± 0.89 ^a^	82.78 ± 8.08 ^a^	10.19 ± 0.70 ^a^	1.56 ± 0.14 ^a^
	JSJ17	46.32 ± 1.02 ^a^	18.63 ± 12.04 ^a^	6.89 ± 0.34 ^a^	42.09 ± 4.91 ^a^	7.80 ± 0.99 ^ab^	80.40 ± 2.44 ^ab^	10.06 ± 0.55 ^ab^	1.28 ± 0.17 ^b^
Flowering stage (90 d)	CK	47.98 ± 0.8 ^d^	25.09 ± 22.67 ^b^	8.17 ± 0.88 ^ab^	54.52 ± 9.91 ^b^	13.83 ± 2.45 ^b^	60.03 ± 8.25 ^b^	7.24 ± 1.06 ^c^	2.39 ± 0.62 ^b^
	CJY1	66.23 ± 2.45 ^b^	28.23 ± 11.53 ^b^	8.07 ± 0.41 ^b^	80.52 ± 12.44 ^a^	20.43 ± 2.23 ^a^	89.61 ± 14.39 ^a^	10.62 ± 1.78 ^b^	2.43 ± 0.62 ^ab^
	CY2	61.93 ± 1.29 ^c^	29.15 ± 17.99 ^b^	8.58 ± 0.53 ^ab^	81.45 ± 9.97 ^a^	21.71 ± 2.39 ^a^	85.02 ± 10.67 ^a^	11.59 ± 1.27 ^ab^	3.31 ± 0.79 ^a^
	JSJ7	68.98 ± 1.71 ^a^	33.62 ± 35.67 ^a^	8.35 ± 0.44 ^ab^	85.77 ± 6.70 ^a^	24.06 ± 2.11 ^a^	96.93 ± 6.69 ^a^	12.86 ± 1.11 ^a^	3.17 ± 0.54 ^ab^
	JSJ17	62.70 ± 2.91 ^c^	27.33 ± 23.96 ^b^	8.91 ± 0.15 ^b^	91.83 ± 14.06 ^a^	24.16 ± 3.84 ^a^	102.01 ± 17.21 ^a^	12.60 ± 2.20 ^ab^	3.26 ± 0.61 ^ab^

Note: Data are presented as mean ± standard error; different lower-case letters in the same column indicate significant differences among the treatments (*p* < 0.05). Treatments CJY1, CY2, JSJ7, JSJ17 are refer to the plants inoculated with the IAA-producing endophytic stains CJY1 (*Bacillus oryzaecorticis*), CY2 (*Pseudomonas psychrotolerans*), JSJ7, and JSJ17 (*Enterobacter cloacae*). CK is untreated.

**Table 3:** Effects of inoculation of IAA-producing endophytic bacteria on the flowers and yield of tea chrysanthemum’.

	Flower					
Treatment	Diameter (mm)	Inflorescence Number	Fresh wt (g)	Fresh wt Single Flower/g	Fresh wt Per Plant/g	Estimated Yield Per mu/kg
CK	44.84 ± 1.20 ^b^	49.17 ± 4.14 ^c^	1.32 ± 0.09 ^c^	0.18 ± 0.01 ^b^	94.90 ± 5.46 ^b^	758.20 ± 43.72 ^b^
CJY1	49.77 ± 1.40 ^a^	79.67 ± 10.84 ^ab^	2.00 ± 0.12 ^b^	0.26 ± 0.02 ^a^	159.33 ± 21.68 ^a^	1274.68 ± 173.48 ^a^
CY2	50.40 ± 0.73 ^a^	73.67 ± 4.96 ^b^	1.94 ± 0.11 ^b^	0.26 ± 0.01 ^a^	142.91 ± 9.61 ^a^	1143.32 ± 76.91 ^a^
JSJ7	50.36 ± 0.84 ^a^	85.17 ± 5.02 ^a^	1.91 ± 0.05 ^b^	0.26 ± 0.01 ^a^	165.92 ± 6.19 ^a^	1325.50 ± 77.14 ^a^
JSJ17	51.11 ± 0.79 ^a^	74.33 ± 11.00 ^ab^	2.14 ± 0.10 ^a^	0.27 ± 0.01 ^a^	171.07 ± 17.71 ^a^	1366.84 ± 203.93 ^a^

Note: Data are presented as mean ± standard error; different lower-case letters in the same column indicate significant differences among the treatments (*p* < 0.05); estimated yield per mu/kg = (flower yield/plant (g) × 12 plants per m^2^ × 666.67)/1000.

## Data Availability

Not applicable.
